# Supplementary material for: Post-exposure protection of SARS-CoV-2 lethal infected K18-hACE2 transgenic mice by neutralizing human monoclonal antibody
Source: Nat Commun. 2021 Feb 11;12:944. doi: 10.1038/s41467-021-21239-8 (PMC7878817; doi:10.1038/s41467-021-21239-8)
Supplement: Supplementary file 1 — Supplementary Information [file 41467_2021_21239_MOESM1_ESM.pdf]

Supplementary Information

Post-exposure protection of SARS-CoV-2 lethal infected K18-hACE2 transgenic mice by neutralizing human monoclonal antibody

Ronit Rosenfeld*, Tal Noy-Porat, Adva Mechaly, Efi Makdasi, Yinon Levy, Ron Alcalay, Reut Falach, Moshe Aftalion, Eyal Epstein, David Gur, Theodor Chitlaru, Einat B. Vitner, Sharon Melamed, Boaz Politi, Ayelet Zauberman, Shirley Lazar, Adi Beth-Din, Yentl Evgy, Shmuel Yitzhaki, Shmuel C. Shapira, Tomer Israely and Ohad Mazor*

Israel Institute for Biological Research, Ness-Ziona, Israel

*Address correspondence to:
Ronit Rosenfeld; Ohad Mazor
Israel Institute for Biological Research
Ness-Ziona 74100, Israel
E-mail: ronitr@iibr.gov.il; ohadm@iibr.gov.il

Supplementary material

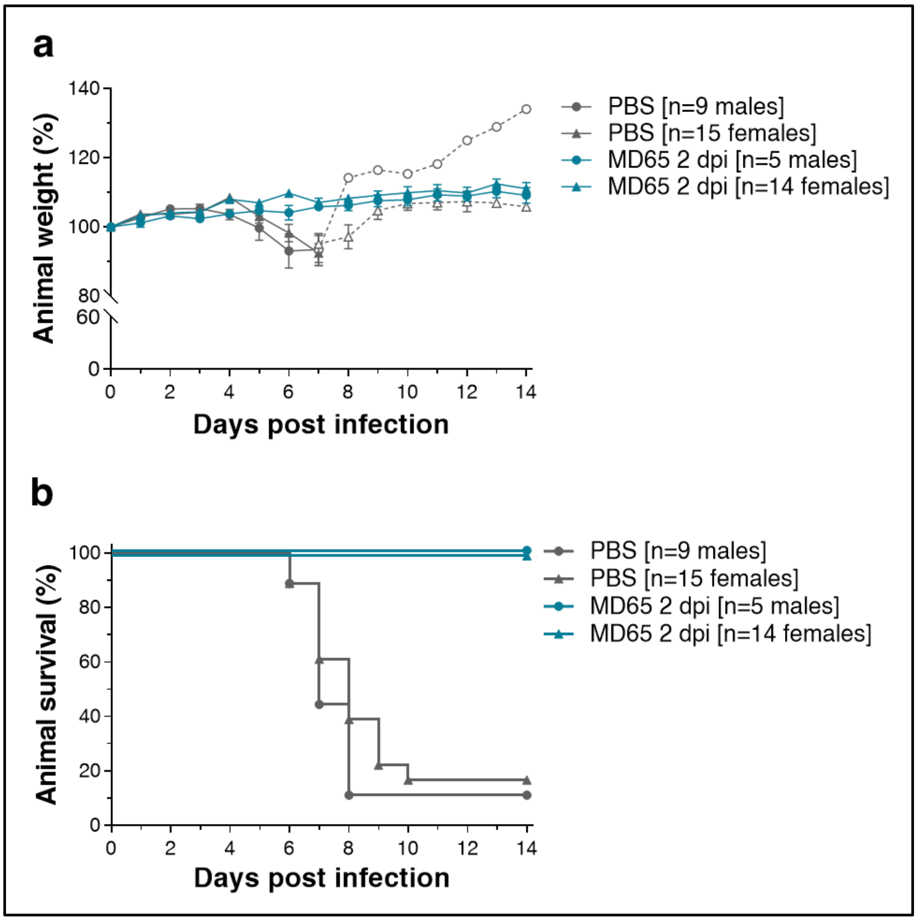

Supplementary Figure 1. Sex-dependent weight loss and survival. Male and female K18-hACE2 mice were administered with PBS or treated with MD65, at day 2 post infection with 200 PFU. a. Body weight profiles. b. Kaplan-Meyer surviving curves. Data represent means ± SEM. Body weight change is displayed as percentage of initial weight. Only data of the first 7 days is presented in the control group exhibiting significant mortality. The weight of the surviving animals is indicated by hollow circles and dashed lines, as indicated.

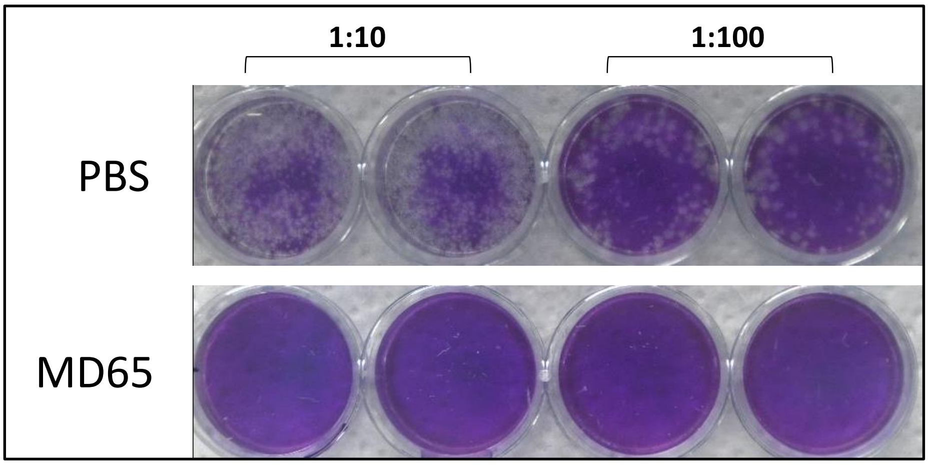

Supplementary Figure 2. Viral load determined by plaque assay of lungs collected from infected mice. Plaque assay was performed to determine the infectious viral load in lung samples 6 dpi from PBS-treated mice (upper raw, n=6) and mice treated 2 dpi with 1 mg MD65 antibody (n=6).

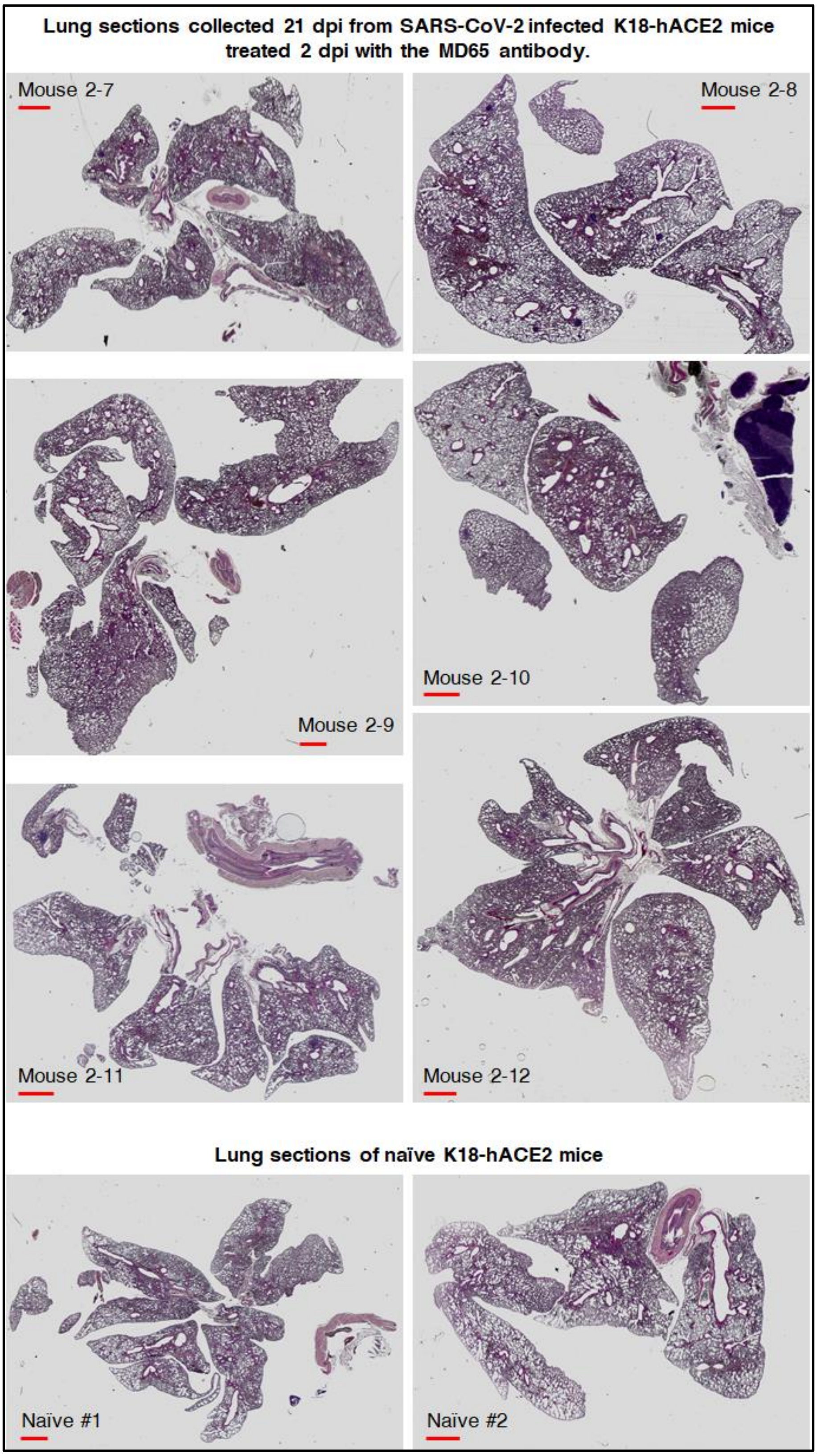

Supplementary Figure 3. Histological analysis of lung sections. Overview of histological sections of lungs, collected at 21 dpi from SARS-CoV-2 independently infected mice that were treated with 1 mg MD65 2 dpi (n=6). Sections of two naïve (uninfected and untreated) mice are also included. Each panel describes a representative micrograph from two analyzed for each sample. Red scale bars=1 mm. Images were obtained using PathScan Enabler IV (Meyer Instruments, USA).

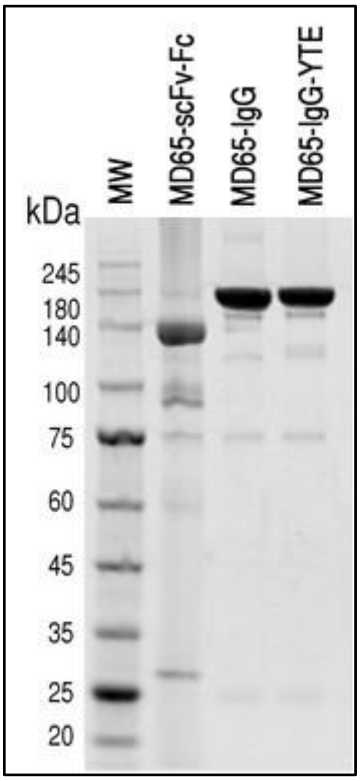

Supplementary Figure 4. **Representative SDS-PAGE analysis of purified MD65 Ab.** MD65 Ab constructed as scFv-Fc, IgG and IgG-YTE were expressed in CHO cells, purified on HiTrap Protein-A column and analyzed by SDS-PAGE. 7 µg of each Ab sample boiled at 100°C for 5 min in sample buffer (Laemmli SB; BIORAD, USA) and was loaded on each lane. Electrophoresis was performed in 1.5 mm thick NuPAGE™ (4-12% Bis-Tris; Invitrogen, USA), run at 80V for 15 min and at 130 V for additional 60 min. The gel was stained with InstantBlue Coomassie (Expedeon, UK). PM2700 MW (Smobio, Taiwan) protein size markers, are indicated. Similar analyses were carried out for each batch (at least 3) of antibodies prior to use.
