# Supplementary material for: Post-exposure protection of SARS-CoV-2 lethal infected K18-hACE2 transgenic mice by neutralizing human monoclonal antibody
Source: Nat Commun. 2021 Feb 11;12:944. doi: 10.1038/s41467-021-21239-8 (PMC7878817; doi:10.1038/s41467-021-21239-8)
Supplement: Supplementary file 3 — Reporting Summary [file 41467_2021_21239_MOESM3_ESM.pdf]

nature research

Corresponding author(s): Ohad Mazor and Ronit Rosenfeld

Last updated by author(s): January, 14th 2021

Reporting Summary

Nature Research wishes to improve the reproducibility of the work that we publish. This form provides structure for consistency and transparency in reporting. For further information on Nature Research policies, see our Editorial Policies and the Editorial Policy Checklist.

Statistics

For all statistical analyses, confirm that the following items are present in the figure legend, table legend, main text, or Methods section.

n/a	Confirmed	
☐	☒	The exact sample size (*n*) for each experimental group/condition, given as a discrete number and unit of measurement
☐	☒	A statement on whether measurements were taken from distinct samples or whether the same sample was measured repeatedly
☒	☐	The statistical test(s) used AND whether they are one- or two-sided<br>*Only common tests should be described solely by name; describe more complex techniques in the Methods section.*
☒	☐	A description of all covariates tested
☒	☐	A description of any assumptions or corrections, such as tests of normality and adjustment for multiple comparisons
☐	☒	A full description of the statistical parameters including central tendency (e.g. means) or other basic estimates (e.g. regression coefficient) AND variation (e.g. standard deviation) or associated estimates of uncertainty (e.g. confidence intervals)
☐	☒	For null hypothesis testing, the test statistic (e.g. *F*, *t*, *r*) with confidence intervals, effect sizes, degrees of freedom and *P* value noted<br>*Give P values as exact values whenever suitable.*
☒	☐	For Bayesian analysis, information on the choice of priors and Markov chain Monte Carlo settings
☒	☐	For hierarchical and complex designs, identification of the appropriate level for tests and full reporting of outcomes
☒	☐	Estimates of effect sizes (e.g. Cohen's *d*, Pearson's *r*), indicating how they were calculated

Our web collection on statistics for biologists contains articles on many of the points above.

Software and code

Policy information about availability of computer code

Data collection	*Provide a description of all commercial, open source and custom code used to collect the data in this study, specifying the version used OR state that no software was used.*
Data analysis	Graphs and statistical analyses were performed in Prism software version 8 (GraphPad Software Inc., USA) and Microsoft Excel 2013. Biolayer interferometry (BLI) experiments results were analyzed using Octet data analysis software 8.1, Fortebio, USA, 2015. Nis-Elements-Br software suite (Nikon, Tokyo, Japan).

For manuscripts utilizing custom algorithms or software that are central to the research but not yet described in published literature, software must be made available to editors and reviewers. We strongly encourage code deposition in a community repository (e.g. GitHub). See the Nature Research guidelines for submitting code & software for further information.

Data

Policy information about availability of data

All manuscripts must include a data availability statement. This statement should provide the following information, where applicable:

- Accession codes, unique identifiers, or web links for publicly available datasets
- A list of figures that have associated raw data
- A description of any restrictions on data availability

GenPept: QHD43416 ORF [https://www.ncbi.nlm.nih.gov/protein/1791269090] was used for SARS-CoV-2 spike protein sequence.
NP_068576.1 ORF [https://www.ncbi.nlm.nih.gov/protein/NP_068576.1] was used for human ACE2 sequence.
Source data are provided with this paper.

Field-specific reporting

Please select the one below that is the best fit for your research. If you are not sure, read the appropriate sections before making your selection.

- [x] Life sciences
- [ ] Behavioural & social sciences
- [ ] Ecological, evolutionary & environmental sciences

For a reference copy of the document with all sections, see nature.com/documents/nr-reporting-summary-flat.pdf

Life sciences study design

All studies must disclose on these points even when the disclosure is negative.

Sample size	No statistical methods were used to predetermine sample size. In vitro experiments were performed with replicates and repeated several times based on previous experience (Noy-Porat et al, Nature Communications, 11:4303) with the protocols and demonstrated high reproducibility.<br>In vivo experiments performed using as a minimum as 4 animal per experimental group and main experiments results were compiled from 3 experiments repeats. Group sizes were selected based on previous experience to obtain statistical significance and reproducibility
Data exclusions	No data were excluded
Replication	All experiments were successfully replicated independently. Exact numbers are indicated in the figures legends.
Randomization	Randomization applied in dividing animals (of each gender) into separate experimental groups. For in vitro studies, all samples, e.g. cells for PRNT, recombinant proteins etc. were allocated randomly into experimental groups.
Blinding	Investigators were not blinded during data collection since the key findings are supported by quantitative measurements (e.g. body weight) that do not rely on subjective judgment for interpretation.

Reporting for specific materials, systems and methods

We require information from authors about some types of materials, experimental systems and methods used in many studies. Here, indicate whether each material, system or method listed is relevant to your study. If you are not sure if a list item applies to your research, read the appropriate section before selecting a response.

Materials & experimental systems

n/a	Involved in the study	
[ ]	[x]	Antibodies
[ ]	[x]	Eukaryotic cell lines
[x]	[ ]	Palaeontology and archaeology
[ ]	[x]	Animals and other organisms
[x]	[ ]	Human research participants
[x]	[ ]	Clinical data
[x]	[ ]	Dual use research of concern

Methods

n/a	Involved in the study	
[x]	[ ]	ChIP-seq
[x]	[ ]	Flow cytometry
[x]	[ ]	MRI-based neuroimaging

Antibodies

Antibodies used	AP-conjugated Donkey anti-human IgG (Jackson ImmunoResearch, USA, Cat# 709-055-149, lot 130049) used at 1:1,000.<br>AP-conjugated Donkey anti-mouse IgG (H+L) minimal cross (Jackson ImmunoResearch, USA, Cat# 715-055-150, lot 142717) used at 1:2,000.
Validation	Antibodies validation was performed by the manufacturer (provided below the validation statements on the manufacturer's website).<br>AP-conjugated Donkey anti-human IgG (Jackson ImmunoResearch, USA, Cat# 709-055-149): Based on immunoelectrophoresis and/or ELISA, the antibody reacts with whole molecule human IgG. It also reacts with the light chains of other human immunoglobulins. No antibody was detected against non-immunoglobulin serum proteins. The antibody has been tested by ELISA and/or solid-phase adsorbed to ensure minimal cross-reaction with bovine, chicken, goat, guinea pig, syrian hamster, horse, mouse, rabbit, rat and sheep serum proteins, but it may cross-react with immunoglobulins from other species.<br>AP-conjugated Donkey anti-mouse IgG (H+L) minimal cross (Jackson ImmunoResearch, USA, Cat# 715-055-150): Based on immunoelectrophoresis and/or ELISA, the antibody reacts with whole molecule mouse IgG. It also reacts with the light chains of other mouse immunoglobulins. No antibody was detected against non-immunoglobulin serum proteins. The antibody has been tested by ELISA and/or solid-phase adsorbed to ensure minimal cross-reaction with bovine, chicken, goat, guinea pig, syrian hamster, horse, human, rabbit and sheep serum proteins, but it may cross-react with immunoglobulins from other species.

Eukaryotic cell lines

Policy information about cell lines

Cell line source(s)	Vero E6 (ATCC® CRL-1586TM) were obtained from the American Type Culture Collection (Summit Pharmaceuticals International, Japan).<br>ExpiCHO-S were purchased from Thermoscientific, USA, Cat# A29127
Authentication	The cell line used was not authenticated
Mycoplasma contamination	Cell lines tested negative for mycoplasma contamination
Commonly misidentified lines<br>(See ICLAC register)	No commonely misidentified cell lines were used in this study

Animals and other organisms

Policy information about studies involving animals; ARRIVE guidelines recommended for reporting animal research

Laboratory animals	Male and female outbred K18-hACE2 transgenic and C57BL/6 mice (Jackson). The age of the K18-hACE2 transgenic mice was 6-10 weeks and that of the C57BL/6 mice 6-8 weeks at the onset of experiments.
Wild animals	No wild animals were used in the experiments.
Field-collected samples	No field collected samples were used in the study.
Ethics oversight	All animal experiments involving SARS-CoV-2 were conducted in a BSL3 facility and treatment of animals was in accordance with regulations outlined in the U.S. Department of Agriculture (USDA) Animal Welfare Act and the conditions specified in the Guide for Care and Use of Laboratory Animals (National Institute of Health, 2011). Animal studies were approved by the local ethical committee on animal experiments (protocol number M-51-20 and M-56-20).

Note that full information on the approval of the study protocol must also be provided in the manuscript.
